# Anti-TIF1 gamma-positive IPAF patient developed stage IVB lung squamous carcinoma in 1 year: a case report

**DOI:** 10.1186/s12890-021-01570-y

**Published:** 2021-06-30

**Authors:** Jiao-jiao Xie, Bin Li, Rui Xu, Xian-zhi Du, Jin-zhi He

**Affiliations:** 1 Department of Respiratory Medicine, The Second Affiliated Hospital of Chongqing Medical University, Chongqing, 400010 People’s Republic of China; 2 Department of Respiratory and Critical Care Medicine, People’s Hospital of Fengjie, Chongqing, 404600 Fengjie People’s Republic of China

**Keywords:** Anti-TIF1γ self-antibody, IPAF, Lung carcinoma, Squamous cell carcinoma, Case report

## Abstract

**Background:**

Patients with connective tissue disease, such as dermatomyositis (DM), and positive anti-TIF1γ self-antibodies are commonly diagnosed with malignant tumors as a comorbidity. The relationship between anti-TIF1γ self-antibodies and existing malignant tumors has been confirmed by several reports. However, interstitial pneumonia with autoimmune features (IPAF) cases with a positive anti-TIF1γ self-antibody developing to solid malignant tumors are rarely reported now.

**Case presentation:**

Herein, we presented an IPAF patient with anti-TIF1γ self-antibodies. No evidence of malignant tumors was found at the initial visit. However, the patient had developed stage IVB lung squamous cell carcinoma at the 1-year follow-up review.

**Conclusions:**

Altogether, this report described a rare case of IPAF patient with anti-TIF1γ self-antibodies developed to advanced lung squamous cell carcinoma in 1 year. The present case highlights more frequent imaging examinations to identify the occurrence of malignant tumors as early as possible in IPAF patients with positive anti-TIF1γ self-antibodies.

## Background

DM patients with positive anti-TIF1γ self-antibodies are commonly diagnosed with malignant tumors as a comorbidity[1–3]. The patient we reported was initially diagnosed as an IPAF case with anti-TIF1γ self-antibody. No evidence of malignant tumors was found. However, through the following visiting, we found the patient had developed stage IVB lung squamous cell carcinoma at the 1-year follow-up review. Recently, more and more rheumatologists and respiratory physicians start to pay more attention to the routine screening of solid tumors in DM patients with anti-TIF1γ self-antibody. However, the follow-up visiting of these patients with no tumor evidence in the initial screening is far from enough. Through this case report, we call for more attention to the follow-up radiologic imaging of these patients.

## Case presentation

**Fig. 1:**
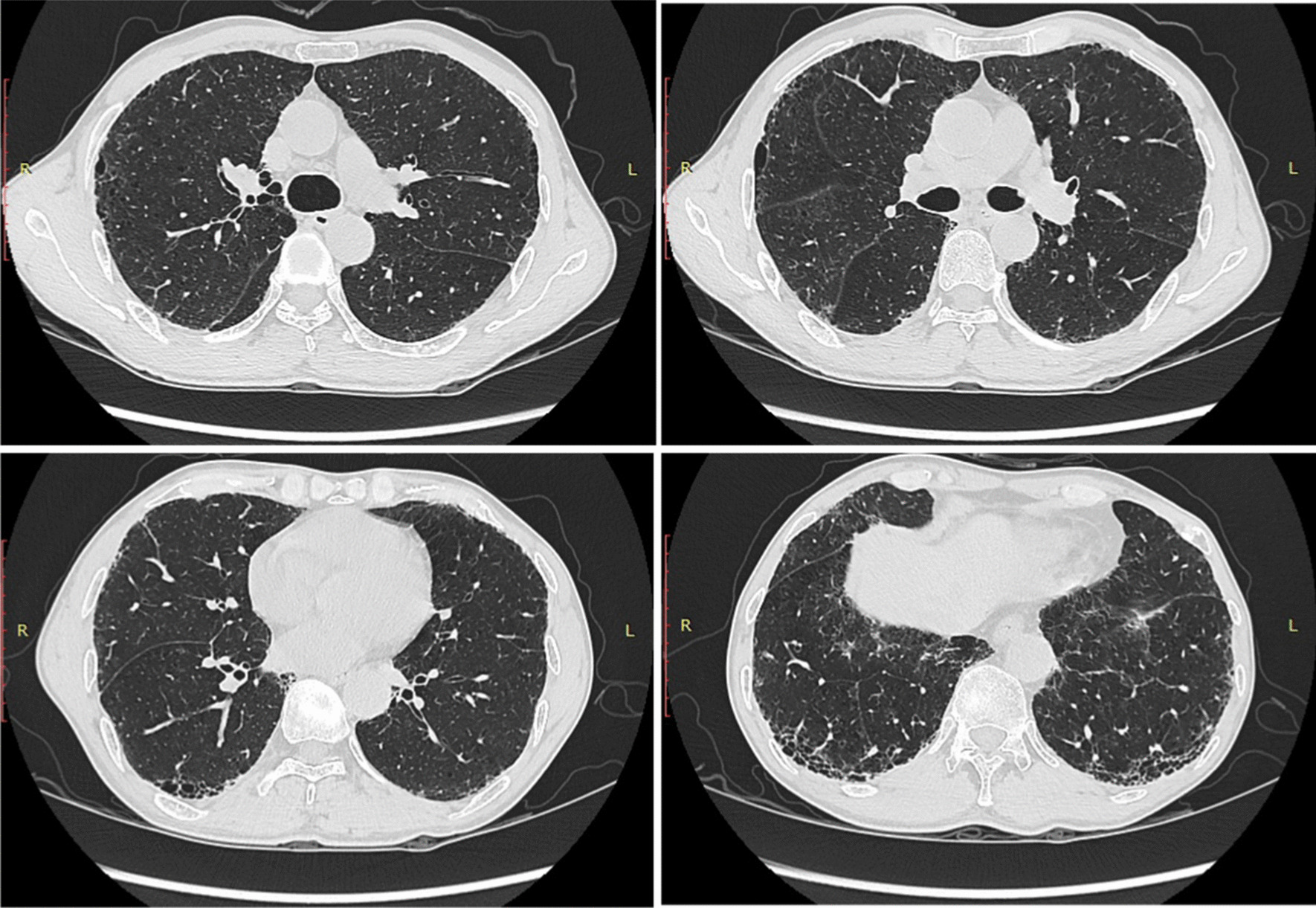
Initial chest HRCT scan performed on July 19th, 2019. Photos indicate interstitial lung inflammation with imaging features of NSIP

A 65-year-old Chinese man presented with gradually worsening cough and shortness of breath for 1 year. Throughout the course of the disease, the patient had no symptoms of hemoptysis or chest pain. He occasionally experienced dry mouth and dry eyes but no itchy skin, desquamation or joint pain. He was admitted to the respiratory department on July 19th, 2019, for further diagnosis and assessment. The patient had normal skin and mucosa, with no rash or scaling. No special past histories and family inherited diseases were reported. Velcro rales could be heard in both lower lungs on physical examination. An initial chest CT scan was taken and indicated interstitial inflammation and fibrosis in the basal segment of the bilateral lower lobe (Fig. 1). In lung function tests, DLCO suggested a moderate diffusion disorder (54.6 % predictive value).

The full blood count, basic metabolic panel, ECG and echocardiography were all normal in this patient. Anti-endothelial cell antibody (AECA) was positive (1:100), but other vasculitis-related self-antibodies were negative. Antinuclear antibody (ANA), anti-CCP, anti-SSA, anti-SSB, Ro-52, anti-nRNP, anti-Sm, anti-Jo1, anti-PM-Scl, anti-Scl-70, and anti-dsDNA antibodies were all negative. However, the myositis antibody panel showed a positive test for the anti-TIF1γ self-antibody.

According to the patient’s positive anti-TIF1γ self-antibody findings and NSIP chest CT manifestation without any clinical signs of DM, such as rash or skin lesions, we diagnosed this patient with IPAF. Subsequently, this patient underwent PET-CT scans and serum tumor marker analysis to search for evidence of potential solid tumors [4]. However, no evidence of any tumor was found. After excluding contraindications, the patient was given oral prednisone (30 mg) once a day and pirfenidone (600 mg) three times per day, respectively.

**Fig. 2:**
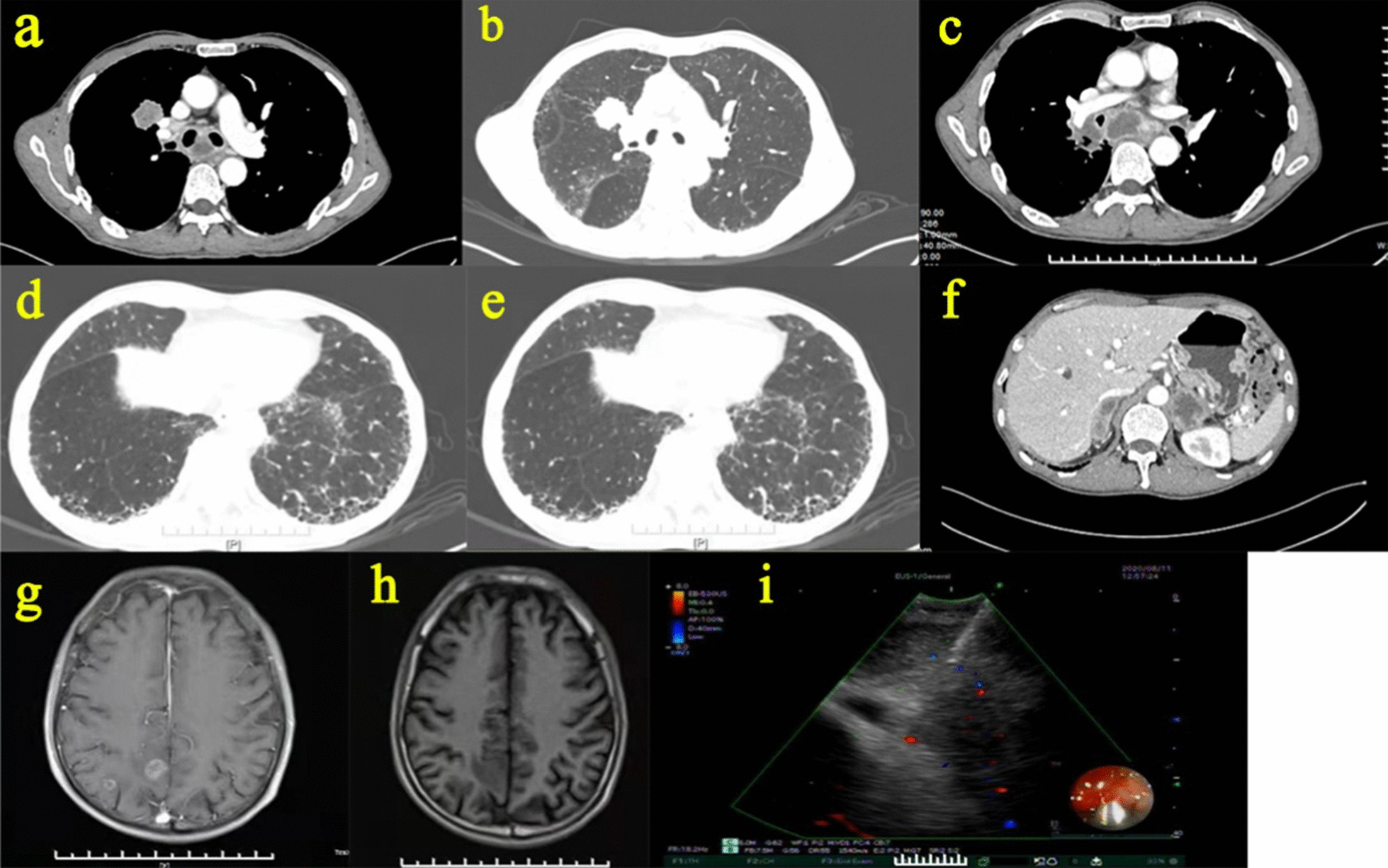
Imaging findings and EBUS-TBNA performed in August 2020 during the second admission of this patient. **a–e** Enhanced chest HRCT showed a newly emerged nodule located on the anterior segment of the right upper lobe that was not present on the HRCT performed in 2019. The mediastinal window suggested enlarged mediastinal and hilar lymph nodes. **f** Enhanced CT of the abdomen revealed bilateral adrenal nodules. **g–h** Brain MRI revealed multiple metastatic nodules in the bilateral cerebral hemispheres. **i** EBUS-TBNA performed on the N_7_ group of enlarged mediastinal lymph nodes

**Fig. 3:**
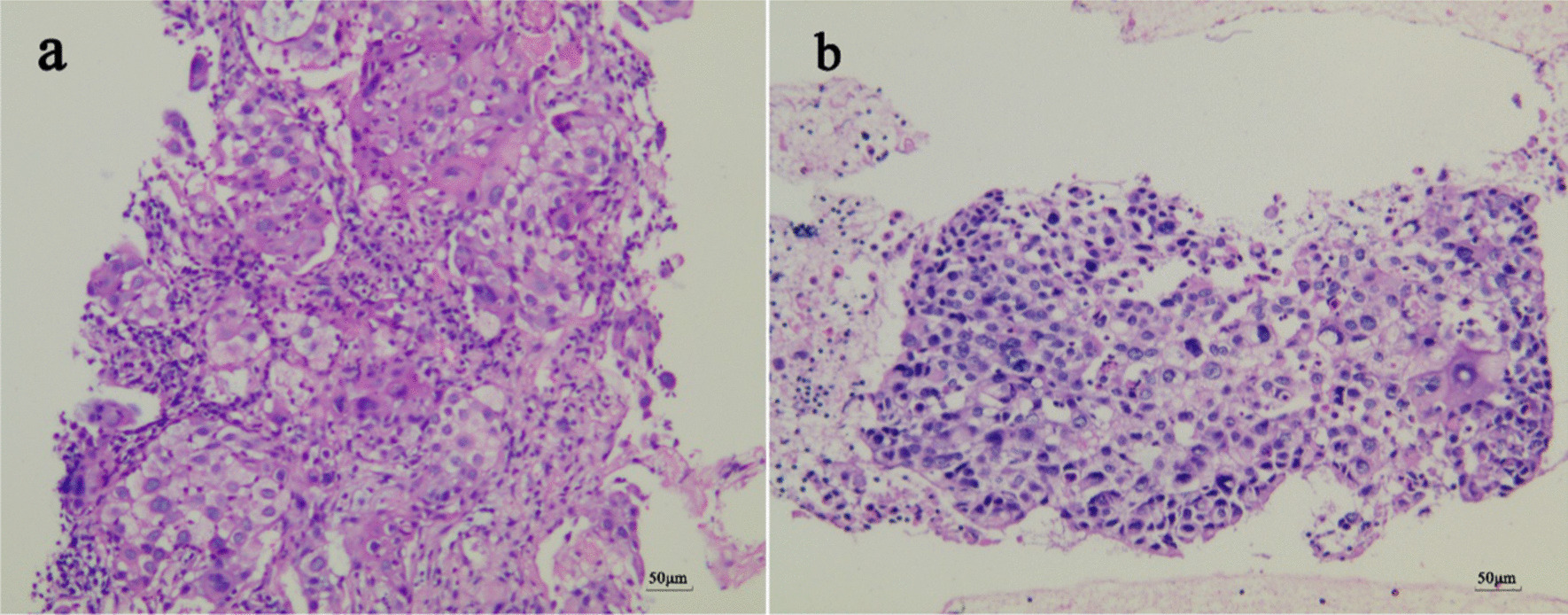
Hematoxylin and eosin sections (original magnification ×200). Slices with H&E staining were observed under the NIKON TE2000-U microscope(10×eyepiece, 20×objective lens). Photos were captured through a CCD digital camera involved in NIKON TE2000-U microscopic platform. Typical photos were recorded by NIKON NIS-Elements software V4.2. **a** CT-guided percutaneous lung biopsy. The tissue cut by fine needle was composed of atypical epithelioid neoplasm cells organized in a squamous carcinoma pattern. **b** EBUS-TBNA. Squamous cell clusters mixed in the mediastinal lymph node tissue of the N7 group aspirated by the fine needle

The patient’s symptoms were not significantly aggravated after these treatments. However, the patient did not return to the hospital for follow-up reviews because of the COVID-19 epidemic in China at the end of 2019. Nearly 1 year later, the patient was admitted to the respiratory department on August 6th, 2020, presenting with aggravated shortness of breath over the previous 3 months. An enhanced chest CT scan showed an irregularly shaped nodule located on the anterior segment of the right upper lobe. Enlarged mediastinal lymph nodes were visible in the right hilum and mediastinum (Fig. 2). Enhanced CT of the abdomen revealed bilateral adrenal nodules, and brain MRI revealed multiple metastatic nodules in the bilateral cerebral hemispheres (Fig. 2). These imaging findings suggested the possibility of malignant lung tumors with multiple metastases. Following CT-guided percutaneous lung biopsy of the right lung nodule and needle aspiration of mediastinal lymph nodes under endobronchial ultrasound-guided transbronchial needle aspiration (EBUS-TBNA), this patient was definitively diagnosed with right lung squamous carcinoma with mediastinal lymph node, brain and adrenal glands metastasis (Fig. 3).

## Discussion and conclusion

Transcriptional intermediary factor 1γ (TIF1γ), an E3 ubiquitin ligase family member, plays a crucial role in regulating TGF-β/Smad signaling in different cellular contexts [5, 6]. Based on its role in this signaling, studies on TIF1γ performed in the last decade have demonstrated a relationship between decreased TIF1γ and cancer [7, 8]. According to past studies, anti-TIF1γ antibodies are detected in approximately 20 % of adult DM patients [9]. Among them, 60–80 % have been diagnosed with malignant tumors as a comorbidity with DM [10]. In our reported case, the patient’s symptoms and physical signs did not meet the criteria for initial diagnosis with DM, so the patient was diagnosed with IPAF. When we first detected the anti-TIF1γ self-antibody, PET-CT scanning and tumor biomarker detection were performed. However, there was no visible evidence of solid tumors. Unfortunately, the patient was diagnosed with stage IVB lung squamous carcinoma at the 1-year follow-up review, despite regular IPAF treatments. Some previous studies have also proposed the importance of follow-up chest CT in assessing the pulmonary status of ILD patients. However, they have not given the exact CT follow-up interval time [11, 12]. Based on this case, we suggest that IPAF patients with positive anti-TIF1γ self-antibodies might need more frequent imaging examinations to identify the occurrence of malignant tumors as early as possible.

## Data Availability

The data that support this case report are available from the corresponding author on reasonable request, since respecting the Ethics Committee to protect patient confidentiality.

## Abbreviations

CTD: Connective tissue disease
DM: Dermatomyositis
IPAF: Interstitial pneumonia with autoimmune features
AECA: Anti-endothelial cell antibody
EBUS-TBNA: Endobronchial ultrasound-guided transbronchial needle aspiration
